# Effects of Sand Dune Stabilization on the Spatial Pattern of *Artemisia ordosica* Population in Mu Us Desert, Northwest China

**DOI:** 10.1371/journal.pone.0129728

**Published:** 2015-06-23

**Authors:** Jiachen Zhang, Yuqing Zhang, Dongqing Fan, Shugao Qin, Xin Jia, Bin Wu, Dong Chen, Hao Gao, Linfeng Zhu

**Affiliations:** 1 Yanchi Research Station, School of Soil and Water Conservation, Beijing Forestry University, Beijing, China; 2 Key Laboratory of Soil and Water Conservation and Desertification Combating of the Ministry of Education, Beijing Forestry University, Beijing, China; York University, CANADA

## Abstract

Vegetation patterns are strongly influenced by sand mobility in desert ecosystems. However, little is known about the spatial patterns of *Artemisia ordosica*, a dominant shrub in the Mu Us desert of Northwest China, in relation to sand fixation. The aim of this study was to investigate and contrast the effects of sand dune stabilization on the population and spatial distribution of this desert shrub. Spatial autocorrelation, semi-variance analysis, and point-pattern analysis were used jointly in this study to investigate the spatial patterns of *A*. *ordosica* populations on dunes in Yanchi County of Ningxia, China. The results showed that the spatial autocorrelation and spatial heterogeneity declined gradually, and the distance between the clustered individuals shortened following sand dune fixation. Seedlings were more aggregated than adults in all stage of dune stabilization, and both were more aggregated on shifting sand dunes separately. Spatial associations of the seedlings with the adults were mostly positive at distances of 0–5 m in shifting sand dunes, and the spatial association changed from positive to neutral in semi-fixed sand dunes. The seedlings were spaced in an almost random pattern around the adults, and their distances from the adults did not seem to affect their locations in semi-fixed sand dunes. Furthermore, spatial associations of the seedlings with the adults were negative in the fixed sand dune. These findings demonstrate that sand stabilization is an important factor affecting the spatial patterns of *A*. *ordosica* populations in the Mu Us desert. These findings suggest that, strong association between individuals may be the mechanism to explain the spatial pattern formation at preliminary stage of dune fixation. Sand dune stabilization can change the spatial pattern of shrub population by weakening the spatial association between native shrub individuals, which may affect the development direction of desert shrubs.

## Introduction

The spatial pattern of a plant population refers to the two-dimensional distribution of individual populations within a certain range [1]. Spatial pattern of populations is the result of long-term interactions between the species and its environment [2, 3], is an important spatial property of plants, and is one of the most fundamental quantitative characteristics. Spatial pattern represents the underlying effect of ecological processes, including biotic interactions, inter- and intra-specific interactions, seed dispersal, vegetation succession, and environmental change [4–6]. Spatial distributions of plant populations can be divided into clumped, random, and regular distribution patterns. Correspondingly, the spatial correlation of populations can be divided into positive, independent, and negative spatial associations [7]. In a population, a clumped distribution and positive spatial association reflect a positive ecological relationship (mutually beneficial), whereas a regular distribution and negative spatial association reflect a negative ecological relationship (mutually exclusive). A random distribution and independent spatial association indicate that there is no clear ecological relationship in a population [8, 9].

Semiarid and arid areas cover over 40% of the earth’s land surface and have been rapidly expanding as a result of climate change and human activities [10]. In the past decades, large bodies of studies have researched the spatial pattern of vegetation in these regions [6, 11, 12]. Many of these studies indicated that spatial distributions and spatial autocorrelation of plants were markedly different between different site conditions, such as different stages of sand burial [6], or different conditions of land patches [5]. Sand dune stabilization, as the most important process in drylands, leads to the changes in site condition. However, the effects of sand dune stabilization on the spatial patterns of plant population are still not clear.

With the continuous development and improvement in methods and tools for spatial data analysis [13–15], spatial auto-correlation, spatial heterogeneity, and point-pattern analysis were widely used to evaluate population spatial patterns [16]. However, every single analysis method has certain drawbacks [17–19]. For example, point-pattern analysis based on individual plant positions, which ignore the plant size, cannot fully reveal the distribution characteristics of plants [20, 21]. Analysis of spatial auto-correlation and heterogeneity can overcome the shortcoming of point-pattern analysis, but cannot discuss the relations between plants with different age classes. Therefore, comprehensive analysis method is conducive to determine the changes of spatial association intensity and internal distribution pattern of population, and to analyze the influence of ecological processes on plant spatial pattern accurately.

*Artemisia ordosica* is one of the dominant shrubs in the semi-arid regions of China, and is an excellent sand-fixing shrub in Mu Us Desert, thereby playing an important role in fixing sand, maintaining biodiversity and ecosystem stability in the region [11]. In this study, spatial auto-correlation, spatial heterogeneity, and spatial point patterns were used jointly for analyses of the spatial patterns of *A*. *ordosica* populations at sites of different sand stabilization stages (i.e., shifting, semi-fixed, and fixed sand dunes) in the Mu Us desert, to reveal the characteristics and variation in the spatial patterns and to understand the distribution strategy of *A*. *ordosica* populations on sand dunes with different sand mobility. The findings will be beneficial to desertification control and vegetation restoration by seeding *A*. *ordosica* in degraded ecosystems.

## Materials and Methods

### Study site and species

The research was conducted on sand dunes at the Yanchi Research Station (106°30′ to 107°41′ E and 37°04′ to 38°10′ N; 1530 m above sea level) of Beijing Forestry University, in Ningxia, Northwest China. The area lies on the southern edge of the Mu Us desert and is characterized by a mid-temperate, semi-arid continental monsoon climate. The mean annual temperature (1954–2004) is 8.1°C and the mean annual precipitation is 287 mm, 62% of which falls from July to September. The mean annual potential evapotranspiration is 2024 mm. The average annual wind speed is 2.8 m/s, which is dominated by a drying westerly and northwesterly wind. The dominant vegetation comprises *A*. *ordosica*, *Hedysarum mongolicum*, *Hedysarum scoparium*, *Salix psammophila*, and other desert shrubs, and *Leymus secalinus*, *Setaria viridis*, *Ixeridium graminifolium*, *Corispermum puberulum*, and other xeric grasses and herbs.

*A*. *ordosica* is a shrub with plumose, linearly lobate leaves [22]. The stem is not obvious. Branches color of adults *A*.*ordosica* is dark gray or dark brown, whereas branches color of seedling *A*. *ordosica* is brown or dark purple. *A*. *ordosica* has tap root system, and its root system is mainly distributed in the upper 30 cm of the sand, while its main roots may reach 1–3 m deep [23]. Recruitment is generally realized by reproduction from seed [24], although plants may occasionally split into clonal fragments [25]. Plants start reproducing at the age of 2–3 years, and reach reproductive peak at the age of 4–7 years.

### Selection of sample plots

In July–September 2013, based on a field survey of the study area, two 30 × 30-m sample plots were selected in shifting, semi-fixed, and fixed sand dunes, respectively. *A*. *ordosica* was the dominant species in all of the six sample plots, which have a relatively flat terrain (data in S1 Table). A coordinate system was established with due west serving as the *x*-axis, due north as the *y*-axis, and the southeast vertex of the plot serving as the origin. The relative locations of the bases of all *A*. *ordosica* plants and their crown diameters in the sample plots were recorded. Table 1 provides basic information of the sample plots.

**Table 1:** Basic information of the sample plots.

No. of sample plot	dune type	Individuals in the plots		No. of individuals (m^-2^)	Vegetation coverage (%)	Biological soil crust coverage (%)	Main companion plants
		seedlings	adults				
P01	S	93	160	0.28	7	0	*Corispermum puberulum*
P02	S	75	196	0.30	10	0	*Corispermum puberulum*
P03	SF	559	447	1.12	25	10	*Corispermum puberulum*
P04	SF	547	449	1.11	27	10	*Corispermum puberulum*
P05	F	21	448	0.52	35	25	*Setaria viridis*, *Ixeridium graminifolium*
P06	F	39	448	0.54	32	45	*Leymus secalinus*, *Setaria viridis*

Notes: S is shifting sand dune, SF is semi-fixed sand dune and F is fixed sand dune.

### Data analysis

The semi-shrub *A*. *ordosica* does not produce notable annual rings, so the age structures need to be distinguished from the morphological characteristics. There was a positive correlation between crowns and heights. Younger *A*. *ordosica* shrubs usually have shorter heights and smaller crowns than older individuals, and averages of their heights and crown is less than 30 cm [5]. Thus, the *A*. *ordosica* individuals were divided into two groups, seedlings and adults, by comparing their averages of heights and crown diameters. Individual plants with an average ($\bar{X}$) less than 30 cm were considered seedlings; larger plants were considered adults.

In this study, covariance (the *I* index proposed by Moran [26]) was used to analyze the auto-correlation of *A*. *ordosica* at distance scale *h*, as in Eq (1):

$$I(h)=\frac{n}{\sum_{i=1}^{n}\sum_{j=1}^{n}W_{ij}}\times \frac{\sum_{i=1}^{n}\sum_{j=1}^{n}W_{ij}(x_{i}-\bar{x})(x_{j}-\bar{x})}{\sum_{i=1}^{n}{\left(x_{i}-\bar{x}\right)}^{2}}$$ (1)

where *W*_*ij*_ is a binomial weight value to indicate whether sample *j* is paired with sample *i* at the distance scale of *h* (1 for pairing and 0 otherwise); *x*_*i*_ and *x*_*j*_ represent the relative coverage of *A*. *ordosica* in samples *i* and *j*; $\bar{x}$ is the arithmetic mean of the relative coverage (given by 1 in this case); *n* is the total number of samples; and *I* is the spatial auto-correlation index, ranging between −1 and 1. An *I* index greater than 0 suggests a positive association, and the higher the value, the stronger the correlation (more aggregated); an *I* index smaller than 0 suggests a negative association; and an *I* index close to 0 suggests a random distribution [27].

Semi-variance can be estimated using Eq (2) [26]:

$$\gamma (h)=\frac{1}{2N(h)}\sum_{i=1}^{N(h)}{\left[Z(x_{i})-Z(x_{i}+h)\right]}^{2}$$ (2)

where *γ* (*h*) is the semi-variance at a distance scale of *h*, *h* is the lag between samples, *N* (*h*) is the number of paired comparisons at lag *h*, and *Z* (*x*_*i*_) and Z(*x*_*i*_ *+h*) are the observed cover values of a given species at locations *x*_*i*_ and *x*_*i*_ *+h*; when semi-variance increases, the spatial association is reduced. The semi-variogram models used in the kriging process need to obey certain numerical properties in order for the kriging equations to be solvable. Therefore, we have choosen the acceptable or licit semivariogram models [27]. The auto-correlation analysis and semi-variance analysis was performed by the tool of GS^+^ for Windows, version 9 (Gamma Design Software, LLC.).

We then analyzed the shrub spatial patterns using the O-ring statistics, which include both univariate and bivariate statistics. O-ring statistics evaluate the expected number of points of a pattern at increasing distances (*t*) from an arbitrary point of a pattern, thereby performing point-pattern analysis [28].

Eq (3) was used to calculate univariate O-ring statistics:

$$O^{\omega }(t)=\frac{\sum_{i=1}^{n}Po\text{int}\left[R_{i}^{\omega }(t)\right]}{\sum_{i=1}^{n}Area\left[R_{i}^{\omega }(t)\right]}$$ (3)

Where $R_{i}^{\omega }(t)$ is an annulus with point *i* as the origin, *t* as the radius, and a width of *w*; $Po\text{int}[R_{i}^{\omega }(t)]$ denotes the number of points in region $R_{i}^{\omega }(t)$; and $Area[R_{i}^{\omega }(t)]$ denotes the area of region $R_{i}^{\omega }(t)$.

The confidence interval lies between the two envelopes, which performed 199 simulations and selected the 10^th^ highest and lowest values. The univariate O-ring statistic (*O*_*11*_ *(t)*) was used to detect the distribution of different age classes (seedlings and adults). Values of *O* ^*ω*^ *(t)* above the estimated upper envelopes indicated that the shrubs were significantly aggregated. Values of *O* ^*ω*^ *(t)* below the estimated lower envelopes indicated that the shrubs were significantly dispersed. Values of the actual statistics between the estimated upper and lower envelopes indicated that the shrubs were randomly distributed [28].

According to the definition of Wiegand and Moloney [14], Eq (4) was used to calculate the bivariate O-ring statistics as follows:

$$O_{12}^{\omega }(t)=\frac{\frac{1}{n1}\overset{n1}{\underset{i=1}{\sum }}Po{\text{int}}_{2}\left[R_{1,i}^{\omega }(t)\right]}{\frac{1}{n1}\overset{n1}{\underset{i=1}{\sum }}Area\left[R_{1,i}^{\omega }(t)\right]}$$ (4)

where *n*_1_ is the number of points of the bivariate statistics object 1 which is represented as adults here; $R_{1,i}^{\omega }(t)$ is an annulus with the *i* ^th^ point of object 1 as the origin, *t* as the radius, and *ω* as the width; $Po{\text{int}}_{2}[R_{1,i}^{\omega }(t)]$ is the point number of bivariate statistics object 2 (represented as seedlings) in region $R_{1,i}^{\omega }(t)$; and $Area[R_{1,i}^{\omega }(t)]$ is the size of the region $R_{1,i}^{\omega }(t)$.

Bivariate O-ring statistics evaluate the expected number of points of seedlings at increasing distances (*t*) from an arbitrary point of adult to determine the spatial association between populations on multiple specified scales. In the case of bivariate analyses, when the actual *O*_*12*_^*ω*^ *(t)* statistics were higher than their estimated upper envelopes, seedlings were considered to be positively associated with adults. When the actual statistics were below their estimated lower envelopes, seedlings were considered to be negatively associated with adults, and values of *O*_*12*_^*ω*^ *(t)* between the estimated upper and lower envelopes indicated that seedlings were neutrally associated with adults [5].

The univariate O-ring statistic (*O* ^*ω*^ *(t)*) was used to detect whether adult shrubs exhibited a tendency to aggregate, and the same analyses were performed for shrub seedlings. In these analyses, the null model was that all individuals followed a heterogeneous Poisson distribution, which removes false aggregation caused by first-order density. We used the bivariate O-ring statistic (*O*_*12*_^*ω*^ *(t)*) to analyze the spatial association of seedlings relative to adult shrubs. For these analyses, the null model was that all adults were position-fixed and all seedlings were randomly spaced relative to adults. That is, the null hypothesis was that the spatial locations of seedlings were not influenced by the locations of adults. Programita, a suitable software package for point-pattern analysis, was used to conduct the O-ring analyses. We performed 199 simulations and selected an estimate of 95% simulation envelopes for both univariate and bivariate O-ring statistics [29].

## Results

### Structure of the *A*. *ordosica* population

Vegetation coverage and biological soil crust coverage were increased following sand dune stabilization. At the shifting sand dune (S) and fixed sand dune (F), *A*. *ordosica* populations exhibited lower plant density (0.28 to 0.54), lower proportion of seedlings (10% to 40%), and higher proportion of adult (60% to 90%) than those of the semi-fixed sand dune (SF) (Table 1). At the SF, *A*. *ordosica* populations exhibited the opposite conditions: higher plant density (1.12), higher proportion of seedlings (>50%), and lower adult proportion (Table 1).

### Spatial autocorrelation and semi-variance analysis of the *A*. *ordosica* population

**Fig 1:**
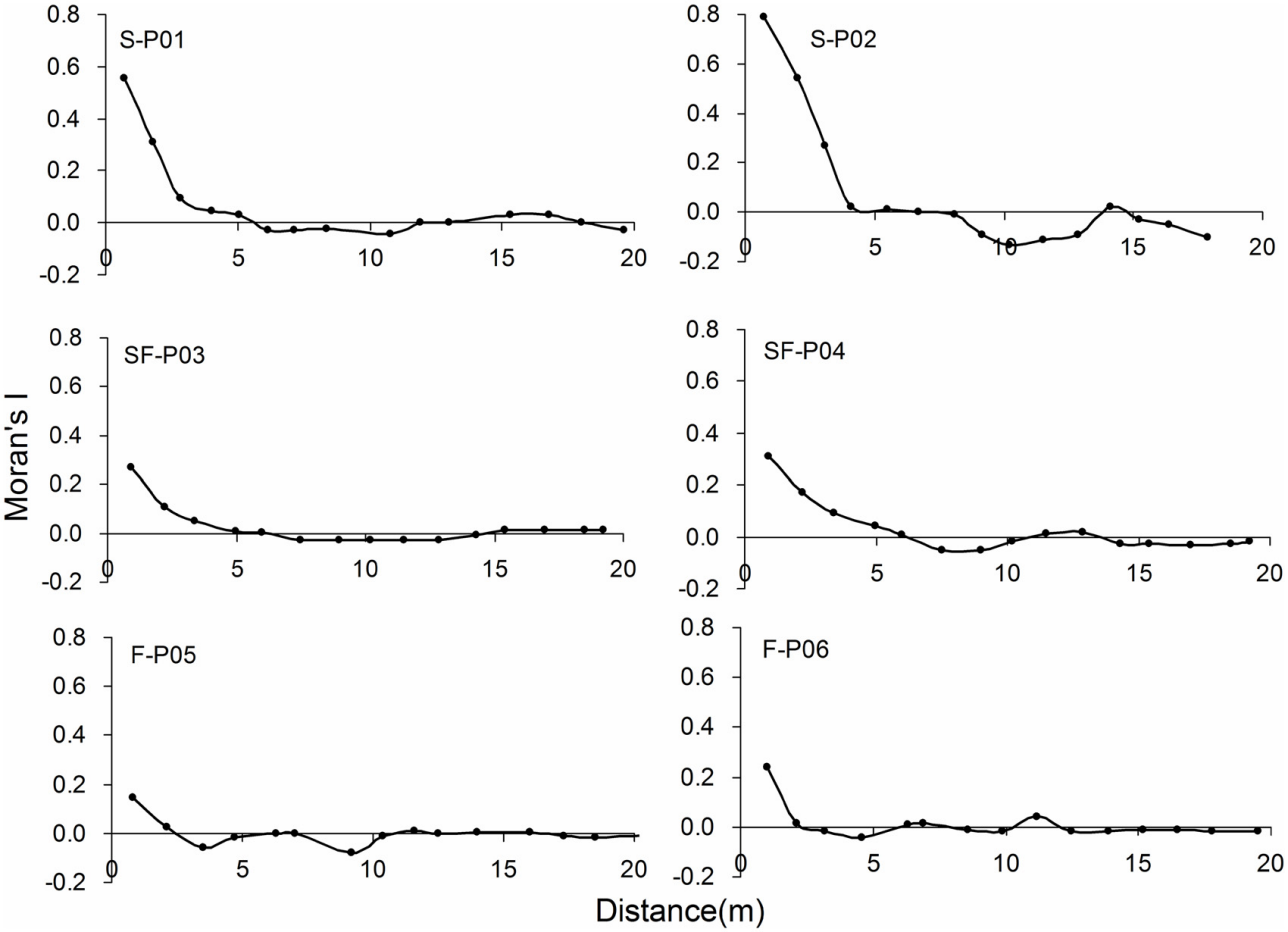
Spatial autocorrelation of *Artemisia ordosica* populations on shifting (S), semi-fixed (SF), and fixed (F) sand dunes.

The spatial autocorrelation of the *A*. *ordosica* population was evaluated across different stages of sand stabilization. In all sample plots, the spatial autocorrelation (*I* value) decreased as the interval distance increased (Fig 1). When comparing different stages of sand dune stabilization, Moran’s *I* values showed a trend of shifting as follows: shifting sand dune > semi-fixed sand dune > fixed sand dune. The range of significant spatial correlation was 0–5 m for shifting and semi-fixed sand dunes, and was 0–2 m for fixed sand dunes.

Semi-variance analysis showed that for all sample plots of *A*. *ordosica*, the largest analysis scale was about 20 m (Table 2). In all six plots studied, the ratio of nugget to sill was less than 50%. The sill represents the semi-variance limit when the interval distance is greater than the range, and its variation is caused by the combined effects of plot randomness and spatial autocorrelation, whereas the nugget represents the variation caused by randomness, and the ratio of nugget to sill represents the proportion of random variation [30, 31]. The range represents the size of the scope of spatial correlation when the variables within the range are spatially correlated. The results showed that the range gradually decreased from shifting to semi-fixed and fixed sand dunes as well as the ratio of nugget to sill showed the same trend.

**Table 2:** Semi-variance analysis of *A*. *ordosica* populations in the sample plots.

No. of sample plot	Optimal model	R²	Range (m)	Nugget	Sill	Nugget/Sill	Analysis scale (m)
P01	Gaussian	0.780	4.82	451	1816	0.2480	19.17
P02	Gaussian	0.915	4.99	571	2127	0.2680	18.29
P03	Exponential	0.955	4.68	148	1506	0.0980	20.37
P04	Exponential	0.856	4.51	153	1758	0.0850	19.54
P05	Spherical	0.940	3.65	1	1629	0.0006	20.79
P06	Spherical	0.821	1.90	6	1160	0.0050	19.80

Note: Sill: The semivariance value at which the variogram levels off. Range: The lag distance at which the semivariogram (or semivariogram component) reaches the sill value. Presumably, autocorrelation is essentially zero beyond the range. Nugget: The nugget represents variability at distances smaller than the typical sample spacing, including measurement error. The spherical model actually reaches the specified sill value, at the specified range. The exponential and Gaussian approach the sill asymptotically, with the practical range, the distance at which the semivariance reaches 95% of the sill value.

### Spatial point-pattern analysis of the *A*. *ordosica* population

**Fig 2:**
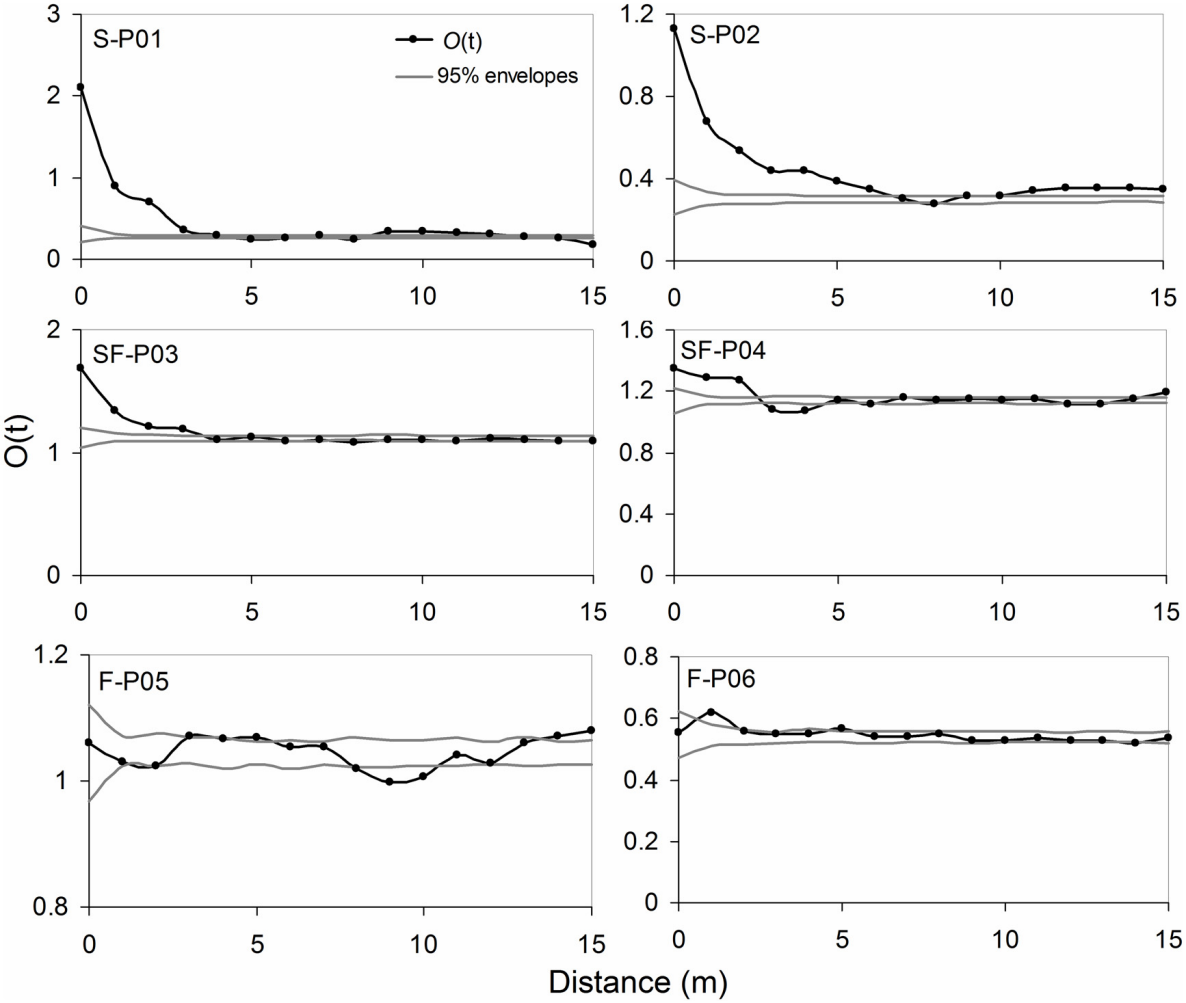
*O(t)* functions of *Artemisia ordosica* population on shifting (S), semi-fixed (SF), and fixed (F) sand dunes.

The spatial patterns of *A*. *ordosica* populations showed a clustered distribution at a distance of 0–6 m on plots of shifting sand dunes, a clustered distribution at a distance of 0–3.5 m on plots of semi-fixed sand dunes, and a clustered distribution within 2 m and random or uniform distribution at a distance of 2–20 m on plots of fixed sand dunes (Fig 2).

**Fig 3:**
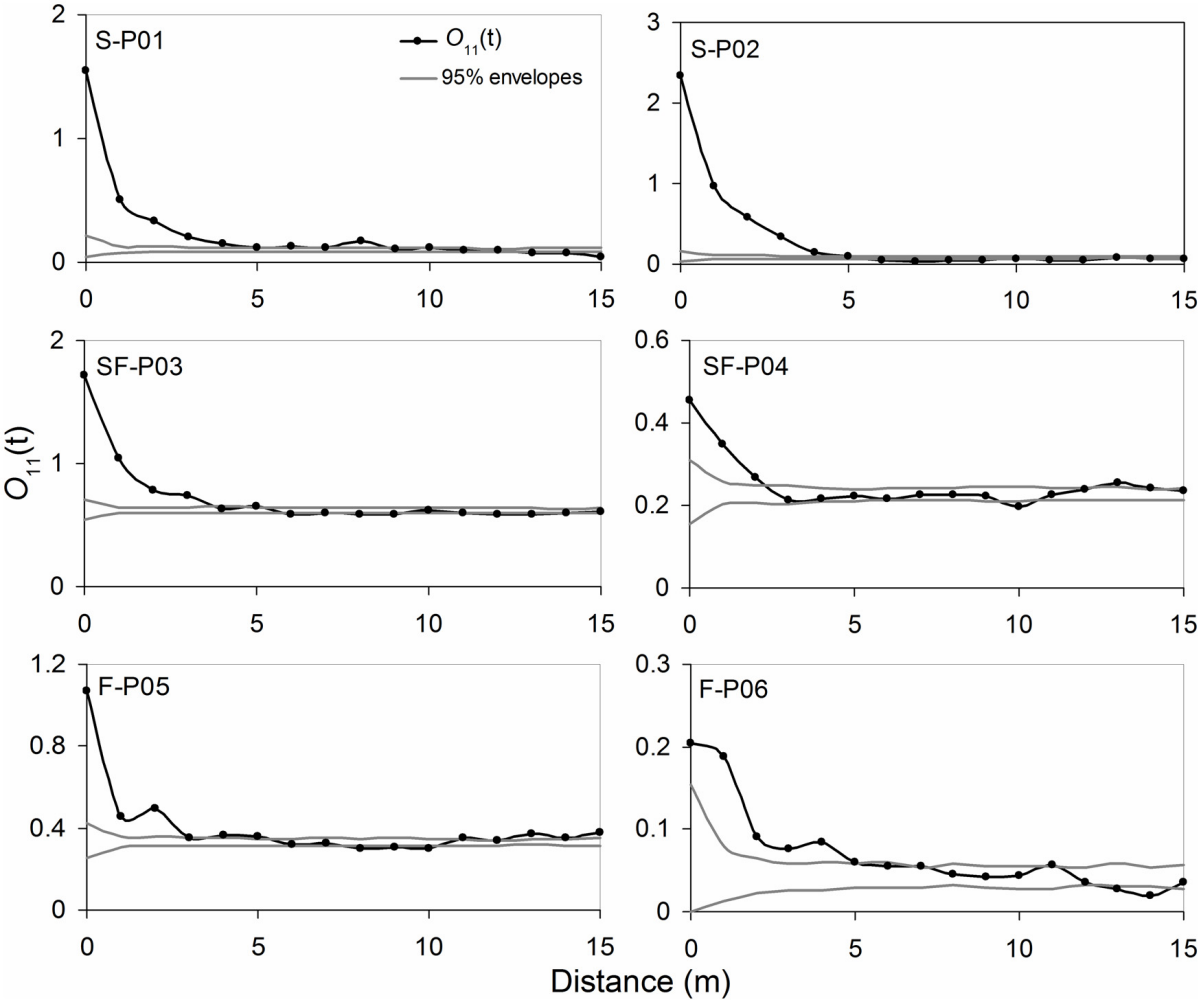
*O(t)* functions of *Artemisia ordosica* seedlings on shifting (S), semi-fixed (S), and fixed (F) sand dunes.

On the shifting sand dunes, the seedlings showed a significant clustered distribution at a distance of 0–8 m, and a clustered distribution of seedlings was found at a distance of 0–4 m on semi-fixed sand dunes; on fixed sand dunes, a clustered distribution was observed at a distance of 0–5 m. In addition, the degree of aggregation declined as the distance increased in all plots (Fig 3).

**Fig 4:**
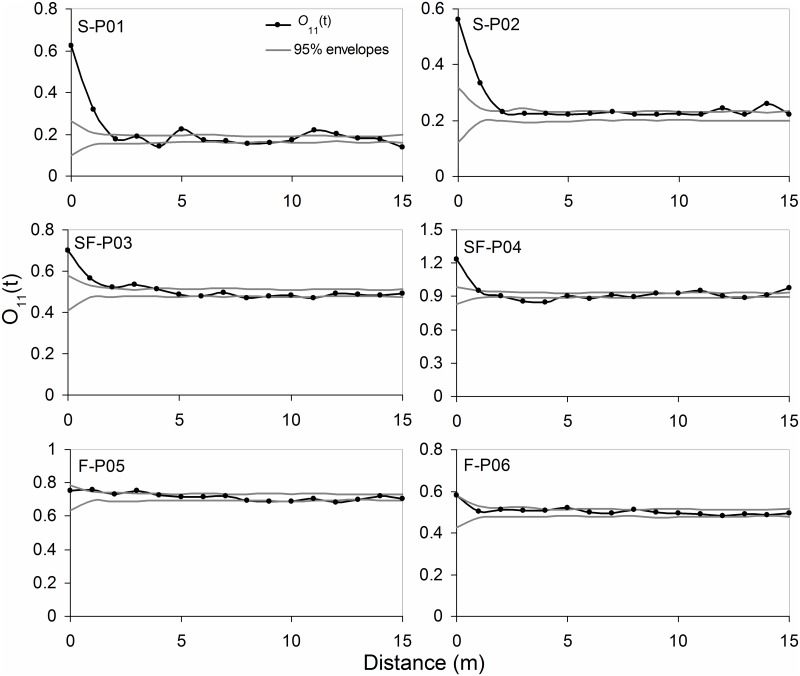
*O(t)* functions of *Artemisia ordosica* adults on shifting (S), semi-fixed (SF), and fixed (F) sand dunes.

Adult plants of *A*. *ordosica* showed a significant clustered distribution on shifting sand dunes at a distance of 0–2 m, and at a distance of 0–1 m on semi-fixed sand dunes (Fig 4). The degree of clustering declined as the distance increased, and this was more prominent on shifting sand dunes than on semi-fixed sand dunes. However, adult plants of *A*. *ordosica* were randomly distributed in the plots on fixed sand dunes.

**Fig 5:**
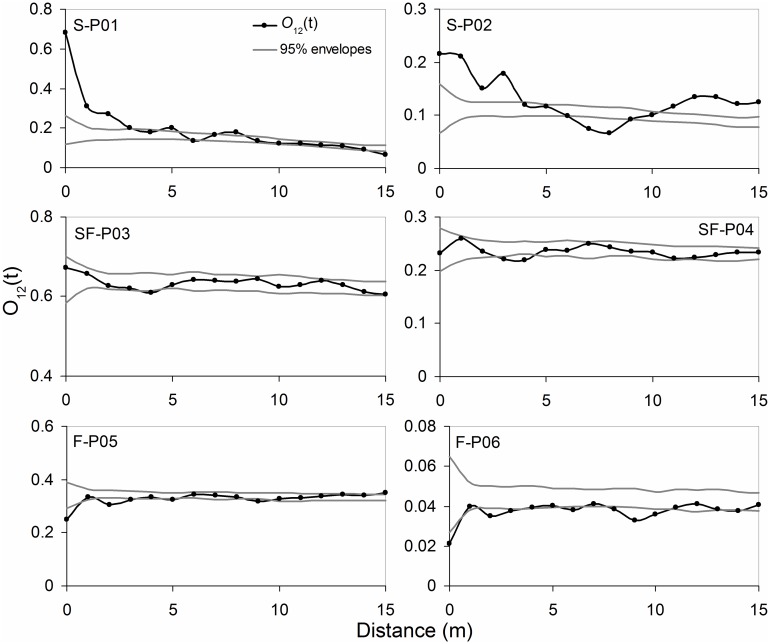
*O*_*12*_ *(t)* functions of *Artemisia ordosica* seedlings with adults on shifting (S), semi-fixed (SF), and fixed (F) sand dunes.

*O*_*12*_ analysis showed that on plots of shifting sand dunes, adult plants and seedlings showed a significant positive spatial correlation at a distance of 0–4m, which indicated that the seedlings were aggregated around the adult plants, whereas *A*. *ordosica* adult plants and seedlings showed a negative spatial correlation on fixed sand plots, suggesting the possibility of competitive exclusion between seedlings and adults (Fig 5). On the semi-fixed sand plots, adult plants and seedlings of *A*. *ordosica* were not spatially correlated, and there was no aggregation between the two.

## Discussion

### Changes in spatial autocorrelation and spatial distribution following sand dune stabilization

The interaction between plants and soil environment is a universal ecological relationship for terrestrial plants, we need to find out whether the difference between *A*. *ordosica* population spatial patterns is controlled by dune fixation process. There was a trend of decrease both in spatial autocorrelation and clustering degree of *A*. *ordosica* population (Fig 1, Table 2). The *A*. *ordosica* population exhibited strong spatial autocorrelation and notable clustered distribution on shifting sand dunes, whereas weak spatial autocorrelation and significant uniform distribution was observed on fixed sand dunes (Figs 1 and 2, Table 2). Sand fixation process will significantly change the population spatial autocorrelation and distribution. Strong spatial autocorrelation of *A*. *ordosica* plants indicates that they can form vegetation patches easily [11]. Thus, it can be considered that spatial autocorrelation, patch differentiation, and clumped distribution reflect the same or approximately the same spatial pattern. Li et al. [32] indicated that the invasion of shrubs in grasslands increases the heterogeneity of spatial distribution of primary vegetation and soil resources following dune stabilization. However, results showed that patches were obvious in shifting sand dunes rather than the later stage of dune fixation. Therefore, in addition to soil environment (water and nutrient), there are surely other factors or process dominates the changes of spatial pattern in different dune fixed stage. Sand flow, as restrictive factor, can be a reasonable explanation. The main characteristic of shifting sand dune is low plant coverage and less soil biological crust, so the sand flow can be driven by strong winds easily. A clumped distribution is conducive to play cluster effect on individual growth that could help to resist the invasion of exotic species and the interference of sand flow [33].

As the *A*. *ordosica* population expands, the shrub coverage increases, and a shifting sand dune will become a fixed sand dune. On fixed sand dunes, the high coverage of the *A*. *ordosica* population would lead to intense intraspecific competition [34], which would reduce the positive spatial autocorrelation of the plant population and eventually cause uniform distribution of the population.

### Changes in spatial association following sand dune stabilization

Seedling distribution patterns in plots with different sand stabilization showed that *A*. *ordosica* seedlings clumped within certain ranges (Fig 3). The clustered distribution of plants at small spatial scales is mainly determined by its biological characteristics. Because the space for reproduction and expansion of a plant is usually limited, the seeds are often scattered either under the canopy of the parent plant or in a nearby open space. Hence, seedlings of a plant often aggregate after seed germination [35]. Moreover, seedlings compete relatively moderately for water and other resources, and mutual sheltering is generally required to withstand environmental stress and improve the chances of survival in an arid environment, which also contributes to the aggregated distribution of young individuals [8,36]. With sand fixation, environmental stress gradually decreases, and the range and degree of aggregation of seedlings are correspondingly reduced.

The seedlings were found to be more aggregated over a wider range than were the adult plants in *A*. *ordosica* populations at each stage of sand dune stabilization (Figs 3 and 4). Both in the seedlings and the adult plants, the range and degree of aggregation reduced as the sand dune stabilized. This is because the demand of individual plants for resources increases as *A*. *ordosica* grows, which causes increasing competition between individuals. Meanwhile, as the individual’s resistance to environmental stress improves, their mutual dependence is reduced. Therefore, for adult individuals, both the range and degree of aggregation decrease. As the plant population develops, the spatial patterns gradually transform from an aggregated distribution to a random distribution due to increasing intraspecific competition, and this pattern has been confirmed in different ecosystems [37–39].

The seedlings aggregated around adult plants differently in plots with different sand dune stabilization (Fig 5). On shifting sand dunes, the seedlings aggregated around adult plants within 5 m. This is likely because adult plants have a sheltering effect (i.e., shading solar radiation and breaking wind by their canopies) on seed germination and seedling growth, contributing to the clumped distribution of seedlings under the canopies of the adult plants. Moreover, the sheltering effect of adult plants on seedlings on shifting sand dunes can reduce the intensity of wind erosion and sand burial. By contrast, on fixed sand dunes there is competitive exclusion between *A*. *ordosica* adult plants and seedlings. Adult *A*. *ordosica* plants are relatively large on fixed sand, requiring a relatively large amount of soil water and nutrients, leading to a competitive relationship with the seedlings whereby the adults prohibit most of the seed germination and seedling growth. Moreover, there are thick biological soil crusts under the shrub canopies, which can limit precipitate water infiltration, making it difficult for seeds to enter the soil and germinate.

A study by McGarigal [40] showed that the internal structure of the plant population can accurately reflect the characteristics of a population’s spatial distribution in response to environmental conditions. Intraspecific relationships have been considered to be important in explaining the spatial distribution of vegetation [33,41], yet they are always referred to as a hypothetical cause. While the results of point-pattern analysis can be used to determine the role of intraspecific relationships, studies of spatial patterns have not taken full advantage of this option [29]. Hence, applying point-pattern analyses in spatial pattern studies can serve to not only assess the condition and characteristics of a population’s distribution, but can also reveal the characteristics of the internal structure of the population and the intraspecific relationships within the population. In this way, the condition and spatial patterns of the *A*. *ordosica* population could be comprehensively and accurately described in plots of different sand dune stabilization stages.

The seedling proportions of *A*. *ordosica* plants in populations varied in different stages of sand dune stabilization. The number of *A*. *ordosica* plants and the average crown diameter were relatively small on shifting sand dunes, while on semi-fixed sand dunes, the *A*. *ordosica* population developed rather well, showing high seedling proportions. On shifting and semi-fixed sand dunes, *A*. *ordosica* populations were in the growth stage, whereas on fixed sand dunes, the *A*. *ordosica* plants were rather uniformly distributed, with only a few sporadic seedlings, and the populations were in degrade stage [42] (Fig 1). The reason may be that the sand-fixing shrubs improved the topsoil texture and nutrient, thus creating conditions for the formation of biological soil crusts. The formation and development of biological soil crusts on the fixed sand surface would then inhibit the infiltration of precipitation into the deep soil layer [43, 44], leading to a reduction in soil moisture for the main root distribution layer (40–100 cm) of *A*. *ordosica*. When the soil moisture content of the shrub root distribution layer falls below 1.5%, the growth of *A*. *ordosica* begins to be inhibited, and shrub coverage can only be maintained at less than 10% [43–45]. Another reason for the decline in *A*. *ordosica* populations on fixed sand dunes may be related to wind erosion and sand burial. In the shifting and semi-fixed sand dunes, these factors might be beneficial to individual growth and population development of *A*. *ordosica* [46]. However, when the vegetation coverage exceeds 50%, the population of *A*. *ordosica* declines because of intense intraspecific competition and insufficient regeneration of seedlings, and *A*. *ordosica* may be replaced by other plant species gradually.

## Conclusion

In the Mu Us desert, the individual of *A*. *ordosica* population distributed differently on dunes with different sand mobility. The degree of aggregation of *A*. *ordosica* individuals was declined as the dunes changed from shifting to fixed conditions, including seedlings and adults. Seedlings were more aggregated than adults in all stage of dune stabilization, and both were more aggregated on shifting sand dunes separately. The association between seedlings and adults declined with the sand dune becoming more fixed. It releases that sand stabilization is an important factor affecting the spatial distribution of *A*. *ordosica* population in the Mu Us desert. These findings suggest that, strong association between individuals may be the mechanism to explain the spatial pattern formation at preliminary stage of dune fixation. Sand dune stabilization can change the spatial pattern of shrub population by weakening the spatial association between native shrub individuals, which may affect the development direction of desert shrubs.

## Supporting Information

S1 Fig Location of the six study sites in Yanchi Research Station (Ningxia, Northwest China). (DOC)

S1 Table Description of main characteristics of the six study sites located in shifting sand dune (S), semi-fixed sand dune (SF) and fixed sand dune (F) of Yanchi Research Station. (DOC)

S2 Table Data of relative locations of all *A*. *ordosica* plants and their crown diameters in the six study sites located in shifting sand dune (S), semi-fixed sand dune (SF) and fixed sand dune (F) of Yanchi Research Station. (XLS)

S3 Table Method Wiegand-Moloney (ring) with 199 replicates for confidence limits. (DOC)
