# Supplementary material for: Effects of Sand Dune Stabilization on the Spatial Pattern of Artemisia ordosica Population in Mu Us Desert, Northwest China
Source: PLoS One. 2015 Jun 23;10(6):e0129728. doi: 10.1371/journal.pone.0129728 (PMC4477905; doi:10.1371/journal.pone.0129728)

**S1 Figure** Location of the six study sites in Yanchi Research Station (Ningxia, Northwest China). Plots (1-6, in green) were located in southeast of Station.


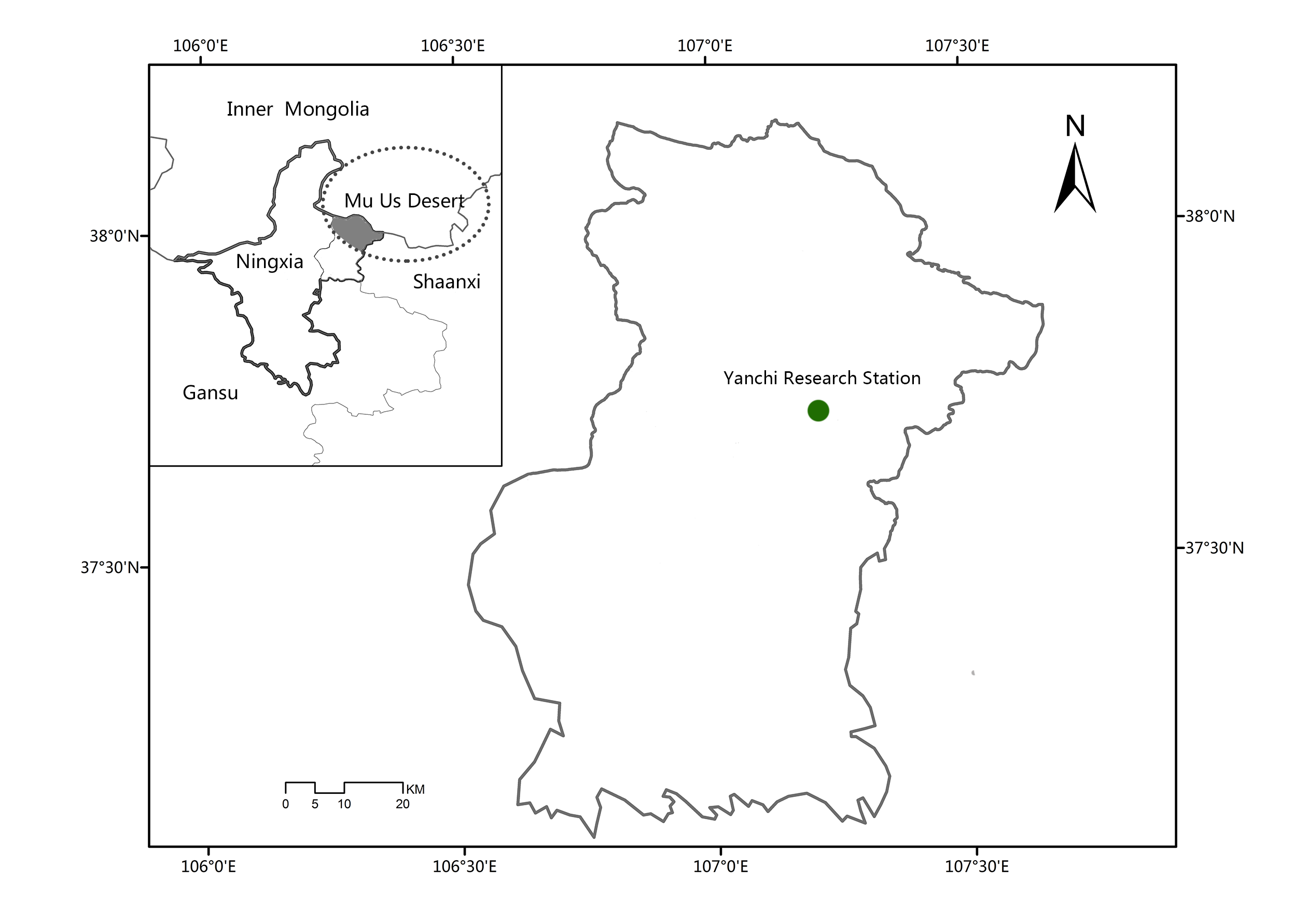


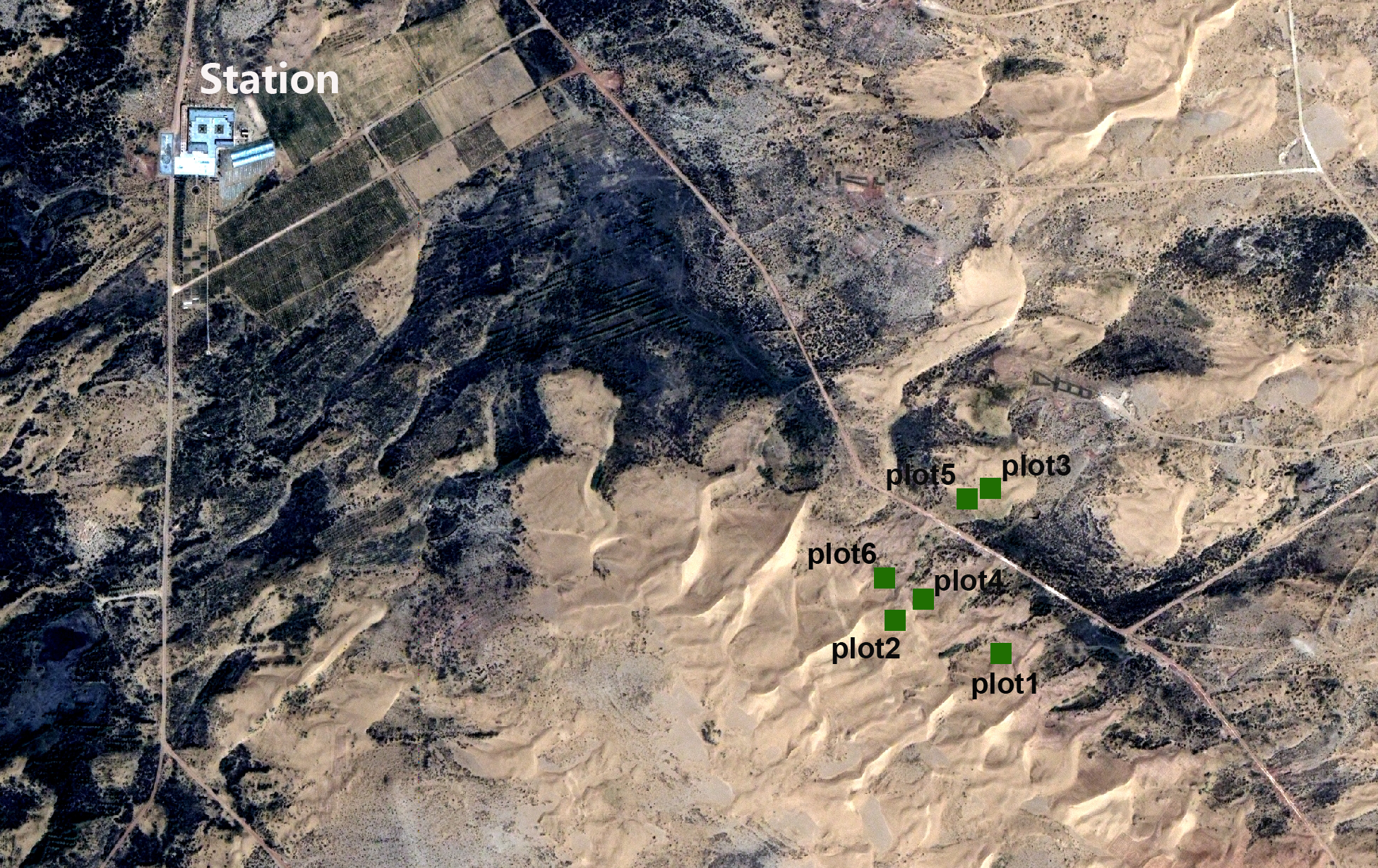

Supplement: S1 Fig — (DOC) [file pone.0129728.s001.doc]
