# Supplementary material for: Effects of Sand Dune Stabilization on the Spatial Pattern of Artemisia ordosica Population in Mu Us Desert, Northwest China
Source: PLoS One. 2015 Jun 23;10(6):e0129728. doi: 10.1371/journal.pone.0129728 (PMC4477905; doi:10.1371/journal.pone.0129728)
Supplement: S1 Table — (DOC) [file pone.0129728.s002.doc]

**S1 Table** Description of main characteristics of the six study sites located in shifting sand dune (S), semi-fixed sand dune (SF) and fixed sand dune (F) of Yanchi Research Station.

| No. of  sample plot | dune type | slope | aspect | altitude | geographic coordinates | |
| --- | --- | --- | --- | --- | --- | --- |
|  |  |  |  |  | latitude (N) | longitude (E) |
| P01 | S | 1° | 307.3° | 1541m | 37°42′11.9″ | 107°14′17.3″ |
| P02 |  | 2° | 311.5° | 1554m | 37°42′10.6″ | 107°14′14.2″ |
| P03 | SF | 2° | 284.2° | 1519m | 37°42′22.9″ | 107°14′13.6″ |
| P04 |  | 0° | 301.7° | 1527m | 37°42′11.9″ | 107°14′9.4″ |
| P05 | F | 2° | 287.1° | 1519m | 37°42′22.9″ | 107°14′13.6″ |
| P06 |  | 0° | 292.3° | 1525m | 37°42′33.2″ | 107°13′27.4″ |
