# Supplementary material for: Effects of Sand Dune Stabilization on the Spatial Pattern of Artemisia ordosica Population in Mu Us Desert, Northwest China
Source: PLoS One. 2015 Jun 23;10(6):e0129728. doi: 10.1371/journal.pone.0129728 (PMC4477905; doi:10.1371/journal.pone.0129728)
Supplement: S3 Table — (DOC) [file pone.0129728.s004.doc]

**S3 Table** Method Wiegand-Moloney (ring) with 199 replicates for confidence limits. Including O (t) for population, O_11_ (t) for seedlings and adults separately, and O_12_ (t) for seedlings with adults.

| Items | | population | | | seedlings | | | adults | | | seedlings & adults | | |
| --- | --- | --- | --- | --- | --- | --- | --- | --- | --- | --- | --- | --- | --- |
| Plot1 | Scale | O11(t) | E11- | E11+ | O11(t) | E11- | E11+ | O11(t) | E11- | E11+ | O12(t) | E12- | E12+ |
|  | 0 | 2.102767 | 0.205534 | 0.403162 | 1.548387 | 0.043011 | 0.215054 | 0.625 | 0.1 | 0.2625 | 0.68125 | 0.11875 | 0.2625 |
|  | 1 | 0.901881 | 0.255983 | 0.312855 | 0.507545 | 0.077778 | 0.131805 | 0.316963 | 0.148637 | 0.204319 | 0.308562 | 0.134895 | 0.204362 |
|  | 2 | 0.699221 | 0.254125 | 0.29816 | 0.331933 | 0.082335 | 0.12426 | 0.174897 | 0.154237 | 0.196651 | 0.270438 | 0.138315 | 0.192403 |
|  | 3 | 0.350834 | 0.261316 | 0.296963 | 0.20057 | 0.081789 | 0.119329 | 0.191196 | 0.156227 | 0.195597 | 0.201096 | 0.142515 | 0.197328 |
|  | 4 | 0.29067 | 0.261341 | 0.295158 | 0.150361 | 0.08296 | 0.118986 | 0.140313 | 0.158811 | 0.193196 | 0.179298 | 0.143321 | 0.193748 |
|  | 5 | 0.252024 | 0.265412 | 0.298099 | 0.116392 | 0.088394 | 0.116146 | 0.222967 | 0.162379 | 0.192445 | 0.201199 | 0.143874 | 0.181716 |
|  | 6 | 0.260504 | 0.263668 | 0.297027 | 0.128591 | 0.086249 | 0.117516 | 0.170346 | 0.161644 | 0.197659 | 0.137535 | 0.140213 | 0.174614 |
|  | 7 | 0.292991 | 0.263804 | 0.294911 | 0.123533 | 0.08629 | 0.117498 | 0.168829 | 0.160262 | 0.191771 | 0.164735 | 0.136332 | 0.169282 |
|  | 8 | 0.244206 | 0.262526 | 0.292295 | 0.174242 | 0.08586 | 0.118366 | 0.156418 | 0.157975 | 0.190958 | 0.178605 | 0.130903 | 0.161807 |
|  | 9 | 0.343276 | 0.262626 | 0.293924 | 0.110597 | 0.087642 | 0.116251 | 0.161037 | 0.162398 | 0.188621 | 0.137421 | 0.126454 | 0.156198 |
|  | 10 | 0.342719 | 0.264642 | 0.296492 | 0.113583 | 0.089952 | 0.1165 | 0.172725 | 0.160807 | 0.189361 | 0.124551 | 0.117368 | 0.143227 |
|  | 11 | 0.324512 | 0.264699 | 0.29413 | 0.100497 | 0.085516 | 0.118163 | 0.220485 | 0.161038 | 0.195461 | 0.120294 | 0.111997 | 0.13529 |
|  | 12 | 0.301996 | 0.266029 | 0.291284 | 0.09638 | 0.089159 | 0.111874 | 0.200767 | 0.167124 | 0.188988 | 0.112846 | 0.105724 | 0.129417 |
|  | 13 | 0.276831 | 0.266213 | 0.298718 | 0.07189 | 0.090721 | 0.117065 | 0.178881 | 0.159134 | 0.190349 | 0.110508 | 0.096291 | 0.121494 |
|  | 14 | 0.253937 | 0.267387 | 0.295088 | 0.073948 | 0.086722 | 0.113779 | 0.175393 | 0.16379 | 0.19052 | 0.093077 | 0.089151 | 0.115474 |
|  | 15 | 0.183236 | 0.262081 | 0.297999 | 0.046248 | 0.088971 | 0.122083 | 0.136646 | 0.160981 | 0.196491 | 0.063923 | 0.082298 | 0.112578 |
|  | | | | | | | | | | | | | |
| Items | | population | | | seedlings | | | adults | | | seedlings & adults | | |
| Plot2 | Scale | O11(t) | E11- | E11+ | O11(t) | E11- | E11+ | O11(t) | E11- | E11+ | O12(t) | E12- | E12+ |
|  | 0 | 1.129151 | 0.228782 | 0.391144 | 2.346667 | 0.026667 | 0.16 | 0.561225 | 0.122449 | 0.316327 | 0.215102 | 0.066327 | 0.158163 |
|  | 1 | 0.674805 | 0.268081 | 0.333333 | 0.964103 | 0.056738 | 0.112478 | 0.332194 | 0.1945 | 0.244032 | 0.211346 | 0.09296 | 0.12987 |
|  | 2 | 0.537938 | 0.275599 | 0.319796 | 0.57367 | 0.064272 | 0.10613 | 0.231277 | 0.199572 | 0.235707 | 0.150531 | 0.099291 | 0.124823 |
|  | 3 | 0.438044 | 0.280183 | 0.319477 | 0.336396 | 0.063987 | 0.102212 | 0.223072 | 0.191202 | 0.242958 | 0.178569 | 0.097625 | 0.124597 |
|  | 4 | 0.437176 | 0.283596 | 0.315392 | 0.138756 | 0.065333 | 0.102094 | 0.224752 | 0.196756 | 0.232604 | 0.119806 | 0.099414 | 0.124904 |
|  | 5 | 0.385092 | 0.283397 | 0.318536 | 0.093842 | 0.070458 | 0.097111 | 0.222139 | 0.196432 | 0.231137 | 0.116041 | 0.098814 | 0.120429 |
|  | 6 | 0.346053 | 0.283458 | 0.318287 | 0.046083 | 0.067677 | 0.097363 | 0.223381 | 0.200685 | 0.233571 | 0.099335 | 0.098575 | 0.119278 |
|  | 7 | 0.30521 | 0.283677 | 0.315616 | 0.024992 | 0.069665 | 0.095164 | 0.229561 | 0.199659 | 0.229789 | 0.073979 | 0.09652 | 0.117398 |
|  | 8 | 0.279588 | 0.286861 | 0.313989 | 0.043698 | 0.069533 | 0.099415 | 0.222336 | 0.202188 | 0.231131 | 0.066338 | 0.094846 | 0.114446 |
|  | 9 | 0.317701 | 0.279542 | 0.313777 | 0.044622 | 0.068798 | 0.100299 | 0.222359 | 0.199597 | 0.234329 | 0.091751 | 0.092041 | 0.113459 |
|  | 10 | 0.313159 | 0.285947 | 0.314673 | 0.059478 | 0.068672 | 0.09509 | 0.223267 | 0.201647 | 0.23202 | 0.101277 | 0.089898 | 0.107725 |
|  | 11 | 0.343434 | 0.28434 | 0.316073 | 0.050407 | 0.064958 | 0.09657 | 0.221653 | 0.199219 | 0.231224 | 0.117026 | 0.087452 | 0.104351 |
|  | 12 | 0.352352 | 0.286374 | 0.313263 | 0.040683 | 0.069872 | 0.095582 | 0.24365 | 0.200223 | 0.230214 | 0.134585 | 0.08547 | 0.102684 |
|  | 13 | 0.356099 | 0.285809 | 0.31588 | 0.081253 | 0.073264 | 0.095631 | 0.221408 | 0.199296 | 0.23401 | 0.134577 | 0.082167 | 0.09849 |
|  | 14 | 0.357017 | 0.287957 | 0.316509 | 0.060296 | 0.06976 | 0.092593 | 0.260272 | 0.199433 | 0.228068 | 0.120894 | 0.078556 | 0.095666 |
|  | 15 | 0.34634 | 0.283639 | 0.319173 | 0.065977 | 0.065805 | 0.098485 | 0.221786 | 0.199099 | 0.235786 | 0.124427 | 0.077057 | 0.09714 |
|  | | | | | | | | | | | | | |
| Items | | population | | | seedlings | | | adults | | | seedlings & adults | | |
| Plot3 | Scale | O11(t) | E11- | E11+ | O11(t) | E11- | E11+ | O11(t) | E11- | E11+ | O12(t) | E12- | E12+ |
|  | 0 | 1.683897 | 1.04175 | 1.198807 | 1.717352 | 0.547406 | 0.70483 | 0.697987 | 0.407159 | 0.577181 | 0.672036 | 0.583893 | 0.700224 |
|  | 1 | 1.345207 | 1.093246 | 1.155229 | 1.047597 | 0.597831 | 0.644195 | 0.565373 | 0.475541 | 0.528368 | 0.658029 | 0.619656 | 0.671598 |
|  | 2 | 1.211846 | 1.09604 | 1.141259 | 0.782039 | 0.599302 | 0.639221 | 0.521656 | 0.471792 | 0.519432 | 0.62707 | 0.617579 | 0.657895 |
|  | 3 | 1.190943 | 1.093819 | 1.138012 | 0.742614 | 0.597208 | 0.641529 | 0.5363 | 0.477296 | 0.508254 | 0.62142 | 0.616941 | 0.657946 |
|  | 4 | 1.106148 | 1.092381 | 1.136958 | 0.632664 | 0.600822 | 0.647754 | 0.513058 | 0.476782 | 0.515361 | 0.609776 | 0.614443 | 0.658462 |
|  | 5 | 1.123182 | 1.096583 | 1.134734 | 0.656248 | 0.601974 | 0.640866 | 0.488817 | 0.475005 | 0.513661 | 0.628237 | 0.620314 | 0.656205 |
|  | 6 | 1.09277 | 1.093869 | 1.139182 | 0.589179 | 0.602377 | 0.644144 | 0.478082 | 0.47775 | 0.511893 | 0.643236 | 0.614157 | 0.661885 |
|  | 7 | 1.100504 | 1.093269 | 1.136635 | 0.594964 | 0.596432 | 0.638196 | 0.497356 | 0.474968 | 0.515722 | 0.640475 | 0.616147 | 0.656065 |
|  | 8 | 1.086428 | 1.097567 | 1.136111 | 0.589569 | 0.599048 | 0.639263 | 0.467569 | 0.479976 | 0.511277 | 0.637043 | 0.614472 | 0.654699 |
|  | 9 | 1.098984 | 1.091843 | 1.142119 | 0.581858 | 0.596205 | 0.641019 | 0.479816 | 0.474592 | 0.512975 | 0.64456 | 0.614699 | 0.651777 |
|  | 10 | 1.104399 | 1.089984 | 1.138272 | 0.615452 | 0.599169 | 0.637476 | 0.482307 | 0.475671 | 0.510074 | 0.624468 | 0.607591 | 0.654442 |
|  | 11 | 1.096588 | 1.090197 | 1.136629 | 0.598524 | 0.594064 | 0.636193 | 0.470949 | 0.47565 | 0.511306 | 0.629308 | 0.61077 | 0.650558 |
|  | 12 | 1.109191 | 1.092409 | 1.135797 | 0.588822 | 0.601704 | 0.638228 | 0.490122 | 0.477129 | 0.511995 | 0.639289 | 0.607696 | 0.643868 |
|  | 13 | 1.099152 | 1.096269 | 1.138251 | 0.587329 | 0.602555 | 0.640694 | 0.485006 | 0.478274 | 0.511299 | 0.62962 | 0.607468 | 0.643147 |
|  | 14 | 1.087801 | 1.095493 | 1.133628 | 0.600572 | 0.600018 | 0.634276 | 0.481328 | 0.479623 | 0.509864 | 0.612589 | 0.604295 | 0.637141 |
|  | 15 | 1.089811 | 1.094644 | 1.135163 | 0.607137 | 0.598746 | 0.637555 | 0.490467 | 0.474171 | 0.511859 | 0.606226 | 0.603761 | 0.637533 |
|  | | | | | | | | | | | | | |
| Items | | population | | | seedlings | | | adults | | | seedlings & adults | | |
| Plot4 | Scale | O11(t) | E11- | E11+ | O11(t) | E11- | E11+ | O11(t) | E11- | E11+ | O12(t) | E12- | E12+ |
|  | 0 | 1.349027 | 1.05642 | 1.215953 | 0.456311 | 0.15534 | 0.31068 | 1.23601 | 0.829684 | 0.982968 | 0.231144 | 0.19708 | 0.278589 |
|  | 1 | 1.29219 | 1.113843 | 1.171753 | 0.348495 | 0.203085 | 0.258106 | 0.950968 | 0.885618 | 0.949833 | 0.259081 | 0.217635 | 0.264202 |
|  | 2 | 1.274573 | 1.116279 | 1.16319 | 0.268568 | 0.204901 | 0.248545 | 0.906089 | 0.891581 | 0.940564 | 0.234778 | 0.224152 | 0.255777 |
|  | 3 | 1.077001 | 1.12223 | 1.163673 | 0.214385 | 0.202695 | 0.249295 | 0.85472 | 0.888936 | 0.934424 | 0.220888 | 0.224703 | 0.253533 |
|  | 4 | 1.070005 | 1.119359 | 1.163437 | 0.217641 | 0.210902 | 0.242215 | 0.850352 | 0.88912 | 0.933868 | 0.219595 | 0.230584 | 0.255093 |
|  | 5 | 1.138507 | 1.121737 | 1.159973 | 0.222222 | 0.209984 | 0.23954 | 0.902787 | 0.891007 | 0.92976 | 0.237834 | 0.225815 | 0.253054 |
|  | 6 | 1.115295 | 1.121796 | 1.162673 | 0.216905 | 0.212295 | 0.243207 | 0.881268 | 0.892342 | 0.934141 | 0.236213 | 0.226434 | 0.255445 |
|  | 7 | 1.160255 | 1.119845 | 1.159678 | 0.227317 | 0.212722 | 0.24163 | 0.910982 | 0.889916 | 0.935468 | 0.249658 | 0.222971 | 0.252804 |
|  | 8 | 1.140273 | 1.121636 | 1.157352 | 0.226382 | 0.214217 | 0.241595 | 0.896038 | 0.890071 | 0.932695 | 0.243835 | 0.227025 | 0.254379 |
|  | 9 | 1.15047 | 1.121102 | 1.161603 | 0.221535 | 0.210328 | 0.244893 | 0.924936 | 0.889095 | 0.933594 | 0.235221 | 0.227566 | 0.251807 |
|  | 10 | 1.143076 | 1.121586 | 1.158315 | 0.198177 | 0.208894 | 0.245161 | 0.923752 | 0.890241 | 0.930844 | 0.233911 | 0.221053 | 0.247735 |
|  | 11 | 1.153739 | 1.119107 | 1.163129 | 0.224888 | 0.212918 | 0.242473 | 0.952775 | 0.891046 | 0.936172 | 0.222015 | 0.218911 | 0.245162 |
|  | 12 | 1.11896 | 1.120262 | 1.160196 | 0.237372 | 0.214302 | 0.242122 | 0.901831 | 0.889605 | 0.930449 | 0.223695 | 0.220492 | 0.244998 |
|  | 13 | 1.11961 | 1.120723 | 1.159645 | 0.25423 | 0.21174 | 0.243562 | 0.885719 | 0.89246 | 0.931567 | 0.229076 | 0.217437 | 0.244424 |
|  | 14 | 1.148876 | 1.120853 | 1.161238 | 0.242118 | 0.212489 | 0.239787 | 0.913196 | 0.891398 | 0.928882 | 0.234154 | 0.216739 | 0.243782 |
|  | 15 | 1.193413 | 1.123005 | 1.157167 | 0.235003 | 0.212846 | 0.243391 | 0.972285 | 0.89483 | 0.93221 | 0.232816 | 0.220668 | 0.241967 |
|  | | | | | | | | | | | | | |
| Items | | population | | | seedlings | | | adults | | | seedlings & adults | | |
| Plot5 | Scale | O11(t) | E11- | E11+ | O11(t) | E11- | E11+ | O11(t) | E11- | E11+ | O12(t) | E12- | E12+ |
|  | 0 | 1.06051 | 0.968153 | 1.121019 | 1.070234 | 0.254181 | 0.421405 | 0.751789 | 0.634526 | 0.786936 | 0.251011 | 0.290824 | 0.390358 |
|  | 1 | 1.03047 | 1.023308 | 1.071169 | 0.455516 | 0.308506 | 0.357645 | 0.760365 | 0.692735 | 0.745042 | 0.334178 | 0.324175 | 0.362518 |
|  | 2 | 1.024153 | 1.023725 | 1.0743 | 0.492451 | 0.311742 | 0.357309 | 0.73225 | 0.690487 | 0.739632 | 0.304578 | 0.330042 | 0.358685 |
|  | 3 | 1.071703 | 1.027399 | 1.069543 | 0.350905 | 0.31125 | 0.353823 | 0.753136 | 0.68651 | 0.735101 | 0.324775 | 0.327577 | 0.355481 |
|  | 4 | 1.066292 | 1.019671 | 1.067927 | 0.365394 | 0.310112 | 0.352511 | 0.723165 | 0.695143 | 0.738386 | 0.335342 | 0.32781 | 0.354368 |
|  | 5 | 1.068551 | 1.02662 | 1.062309 | 0.359362 | 0.313227 | 0.343757 | 0.713872 | 0.695687 | 0.732138 | 0.325693 | 0.327251 | 0.351344 |
|  | 6 | 1.05357 | 1.020429 | 1.064046 | 0.322694 | 0.310256 | 0.345186 | 0.713291 | 0.692209 | 0.734979 | 0.343908 | 0.329861 | 0.353372 |
|  | 7 | 1.053945 | 1.025753 | 1.06271 | 0.327521 | 0.315703 | 0.348985 | 0.720099 | 0.693311 | 0.729703 | 0.340795 | 0.325761 | 0.352888 |
|  | 8 | 1.019053 | 1.022411 | 1.069423 | 0.300218 | 0.314254 | 0.342766 | 0.693688 | 0.693043 | 0.732266 | 0.33329 | 0.327568 | 0.35162 |
|  | 9 | 0.997942 | 1.022167 | 1.065356 | 0.308869 | 0.312053 | 0.350377 | 0.686971 | 0.691201 | 0.735364 | 0.318641 | 0.32742 | 0.351767 |
|  | 10 | 1.007375 | 1.024406 | 1.064733 | 0.298553 | 0.315315 | 0.345251 | 0.687597 | 0.690583 | 0.731117 | 0.327484 | 0.319247 | 0.349369 |
|  | 11 | 1.040222 | 1.022764 | 1.068607 | 0.351445 | 0.314304 | 0.345832 | 0.703707 | 0.691682 | 0.738447 | 0.331941 | 0.317762 | 0.346786 |
|  | 12 | 1.028398 | 1.026485 | 1.063419 | 0.341194 | 0.314177 | 0.342125 | 0.682141 | 0.695077 | 0.728814 | 0.33614 | 0.321159 | 0.348352 |
|  | 13 | 1.060607 | 1.022712 | 1.068941 | 0.369609 | 0.316997 | 0.345547 | 0.699522 | 0.697682 | 0.732537 | 0.343473 | 0.320325 | 0.345686 |
|  | 14 | 1.070347 | 1.025663 | 1.062414 | 0.354785 | 0.314884 | 0.345249 | 0.722706 | 0.692236 | 0.728923 | 0.340367 | 0.32102 | 0.343176 |
|  | 15 | 1.079453 | 1.025517 | 1.064024 | 0.377364 | 0.312012 | 0.350454 | 0.706782 | 0.693886 | 0.730527 | 0.349668 | 0.319751 | 0.34405 |
|  | | | | | | | | | | | | | |
| Items | | population | | | seedlings | | | adults | | | seedlings & adults | | |
| Plot6 | Scale | O11(t) | E11- | E11+ | O11(t) | E11- | E11+ | O11(t) | E11- | E11+ | O12(t) | E12- | E12+ |
|  | 0 | 0.552977 | 0.472279 | 0.62423 | 0.205128 | 0 | 0.153846 | 0.579286 | 0.424107 | 0.580357 | 0.021339 | 0.026786 | 0.064732 |
|  | 1 | 0.618596 | 0.50973 | 0.578792 | 0.1875 | 0.013468 | 0.08 | 0.505131 | 0.472263 | 0.52967 | 0.039985 | 0.03793 | 0.052044 |
|  | 2 | 0.55638 | 0.514196 | 0.562764 | 0.090909 | 0.021938 | 0.064846 | 0.512195 | 0.47631 | 0.518519 | 0.035062 | 0.039055 | 0.050324 |
|  | 3 | 0.548024 | 0.519938 | 0.555412 | 0.076285 | 0.025678 | 0.05891 | 0.5091 | 0.475481 | 0.522829 | 0.037821 | 0.038467 | 0.049826 |
|  | 4 | 0.548434 | 0.522621 | 0.565935 | 0.084548 | 0.026506 | 0.060109 | 0.505509 | 0.47735 | 0.513112 | 0.039352 | 0.038903 | 0.050259 |
|  | 5 | 0.564865 | 0.524839 | 0.556967 | 0.059387 | 0.02921 | 0.058158 | 0.5219 | 0.47966 | 0.510034 | 0.040305 | 0.039378 | 0.04908 |
|  | 6 | 0.539972 | 0.519966 | 0.557859 | 0.055808 | 0.029703 | 0.060606 | 0.498795 | 0.477538 | 0.516176 | 0.037883 | 0.03996 | 0.048766 |
|  | 7 | 0.539702 | 0.525073 | 0.558986 | 0.055655 | 0.028941 | 0.054131 | 0.493189 | 0.478115 | 0.516796 | 0.041 | 0.039684 | 0.048437 |
|  | 8 | 0.550502 | 0.521266 | 0.556438 | 0.045381 | 0.032637 | 0.058226 | 0.510834 | 0.481097 | 0.511084 | 0.038489 | 0.039686 | 0.048845 |
|  | 9 | 0.529599 | 0.520766 | 0.559524 | 0.042772 | 0.029248 | 0.055517 | 0.499714 | 0.472435 | 0.514872 | 0.033001 | 0.039157 | 0.048931 |
|  | 10 | 0.529076 | 0.52214 | 0.556409 | 0.043381 | 0.027586 | 0.055109 | 0.493401 | 0.479489 | 0.515475 | 0.035919 | 0.038369 | 0.047168 |
|  | 11 | 0.534599 | 0.522852 | 0.55756 | 0.057236 | 0.028235 | 0.054499 | 0.492317 | 0.479127 | 0.510822 | 0.039448 | 0.038696 | 0.048228 |
|  | 12 | 0.52662 | 0.522441 | 0.55344 | 0.036161 | 0.032529 | 0.053274 | 0.48253 | 0.47897 | 0.512778 | 0.040943 | 0.037245 | 0.047856 |
|  | 13 | 0.529052 | 0.524649 | 0.557891 | 0.028296 | 0.03011 | 0.058114 | 0.490536 | 0.477595 | 0.509988 | 0.038549 | 0.037955 | 0.048459 |
|  | 14 | 0.520706 | 0.524061 | 0.553932 | 0.019858 | 0.030046 | 0.054259 | 0.484664 | 0.481175 | 0.509738 | 0.037606 | 0.038105 | 0.047146 |
|  | 15 | 0.53489 | 0.519979 | 0.559538 | 0.0358 | 0.028261 | 0.057143 | 0.49427 | 0.478537 | 0.51452 | 0.040451 | 0.037754 | 0.046904 |
